# Loss-of-function myostatin mutation increases insulin sensitivity and browning of white fat in Meishan pigs

**DOI:** 10.18632/oncotarget.16822

**Published:** 2017-04-04

**Authors:** Chunbo Cai, Lili Qian, Shengwang Jiang, Youde Sun, Qingqing Wang, Dezun Ma, Gaojun Xiao, Biao Li, Shanshan Xie, Ting Gao, Yaoxing Chen, Jie Liu, Xiaorong An, Wentao Cui, Kui Li

**Affiliations:** ^1^ Institute of Animal Sciences, Chinese Academy of Agricultural Sciences, Beijing, 100193, P. R. China; ^2^ State Key Laboratory of Agro Biotechnology, China Agricultural University, Beijing, 100193, P. R. China; ^3^ College of Animal Medicine, China Agricultural University, Beijing, 100193, P. R. China; ^4^ Institute of Animal Sciences, Qingdao, 266100, P. R. China; ^5^ Department of Bioengineering and Biotechnology, College of Chemical Engineering, Qingdao University of Science and Technology, Qingdao, 266042, P. R. China

**Keywords:** myostatin deficiency, skeletal muscle, fat, insulin sensitivity, irisin

## Abstract

Myostatin-deficient mice showed a remarkable hypertrophy of skeletal muscle, with a decreased fat mass and enhanced insulin sensitivity. Currently, it is unclear if the inhibition of myostatin could be used as an approach to treat human obesity and insulin resistance. In this study, we investigated if the inhibition of porcine myostatin has any effect on fat deposition and insulin sensitivity using genetically engineered Meishan pigs containing a myostatin loss-of-function mutation (*Mstn* ^−/−^ ). Our results indicated that, when compared with wild-type pigs, the amount of subcutaneous fat and leaf fat of *Mstn* ^−/−^ pigs were significantly decreased mainly due to the browning of subcutaneous adipose tissue. Additionally, the serum insulin level decreased and the insulin sensitivity increased significantly in *Mstn* ^−/−^ pigs. Moreover, we found a significant increase in levels of insulin receptor and insulin receptor substrate proteins in skeletal muscle of *Mstn* ^−/−^ pigs, which then activating the insulin signaling pathway. Irisin-mediated regulation is not the only pathway for the activation of insulin signal in *Mstn* ^−/−^ skeletal muscle. This study provides valuable insight for the treatment of human obesity and diabetes mellitus.

## INTRODUCTION

Myostatin is a transcriptional growth factor, also known as growth/differentiation factor 8 (GDF8), which is a member of the transforming growth factor beta (TGFβ) superfamily. Myostatin is expressed mainly in skeletal muscle [1], and a small amount can be detected in fat, brain, heart, liver, and kidney [2, 3]. The main function of myostatin is its negative regulation of skeletal muscle growth and development [4]. *Mstn* ^−/−^ mice have a remarkable increase in skeletal muscle mass and a significant decrease in fat mass when compared with their corresponding wild-type littermates [1]. It has been well established that natural mutations in myostatin can lead to skeletal muscle hypertrophy in species such as cattle [5, 6]. Therefore, myostatin becomes an important target for improving lean meat production in livestock production.

In addition to regulating the growth and development of skeletal muscle, myostatin also plays a regulatory role in fat deposition. It has been observed that myostatin expression in skeletal muscle and adipose tissue is significantly higher in obese mice and obese patients than in normal mice and healthy humans [7–9]. In transgenic myostatin propeptide mice where myostatin activity is inhibited, food-induced obesity was significantly reduced [10]. *Mstn* ^−/−^ mice had a reduced fat deposition compared with wild-type mice, and this phenotype was particularly pronounced with aging [11–13]. The main reason for the decrease in fat deposition in *Mstn* ^−/−^ mice is due to the increased browning of the white adipose tissue [14, 15]. The expression of browning marker genes, such as uncoupling protein 1 (UCP1), peroxisome proliferative activated receptor, gamma, coactivator 1 alpha (PGC-1α), PR domain containing 16 (PRDM16), cell death-inducing DNA fragmentation factor, alpha subunit-like effector A (Cidea), tumor necrosis factor receptor superfamily member 9 (CD137), transmembrane protein 26 (Tmem26), was significantly increased in the white fat of *Mstn* ^−/−^ mice, which subsequently leads to fat consumption [14, 15]. It is thus highly possible that the inhibition of myostatin activity can be used as a new strategy to treat obesity.

Recently, a number of studies have demonstrated that myostatin can also regulate the body's insulin sensitivity in mice. Insulin resistance has been known as one of the key cause for type 2 diabetes, so enhancing the insulin sensitivity could be an effective method to treat type 2 diabetes [16–18]. Compared with normal mice, the expression of myostatin in skeletal muscle increased significantly in diabetic mice [19, 20]. Very interestingly, the same phenomena were also observed in diabetic patients [21, 22]. Inhibition of myostatin activity by myostatin propeptide significantly attenuated insulin resistance in mice fed with a high-fat diet [10, 19]. The insulin sensitivity increased significantly in *Mstn* ^−/−^ mice fed with either normal or high-fat diet [13, 20, 23]. Up to date, the regulatory mechanism of the insulin sensitivity by myostatin remains unclear. Bonala et al. [8] reported that myostatin can promote insulin receptor substrate 1 (IRS1) protein degradation, and thus weakening the activation of insulin signaling pathways, and subsequently resulting in insulin resistance. Dong et al. [24] reported that myostatin inhibition can reduce the expression of proinflammatory factors in adipose tissue, thus enhancing the insulin sensitivity in mice. It is evident that more investigation is required to study the molecular mechanism by which myostatin inhibition regulates the insulin sensitivity *in vivo*.

Currently, most functional studies on myostatin are mainly focused on small animals like mice with very little effort being made to use large mammals such as pigs. Pigs are the closest species similar to humans [25] and can thus be used as an ideal experimental animal model in the research and development of therapeutics to treat human diseases. Recently, our lab had successfully produced myostatin-deficient (*Mstn* ^−/−^) Meishan pigs by using ZFN editing technology [26]. In this study, we investigated the effect of loss-of-function myostatin mutation on fat deposition and insulin sensitivity in pigs. Our findings demonstrate that, for the first time, the *in vivo* inhibition of myostatin activity resulted in significant increases in both insulin sensitivity and fat consumption in *Mstn* ^−/−^ Meishan pigs, and thus provide very useful insight into the use of myostatin inhibitors as an effective alternative approach to treat obesity and type 2 diabetics.

## RESULTS

### An increase in skeletal muscle mass in *Mstn* ^−/−^ Meishan pigs

**Figure 1:**
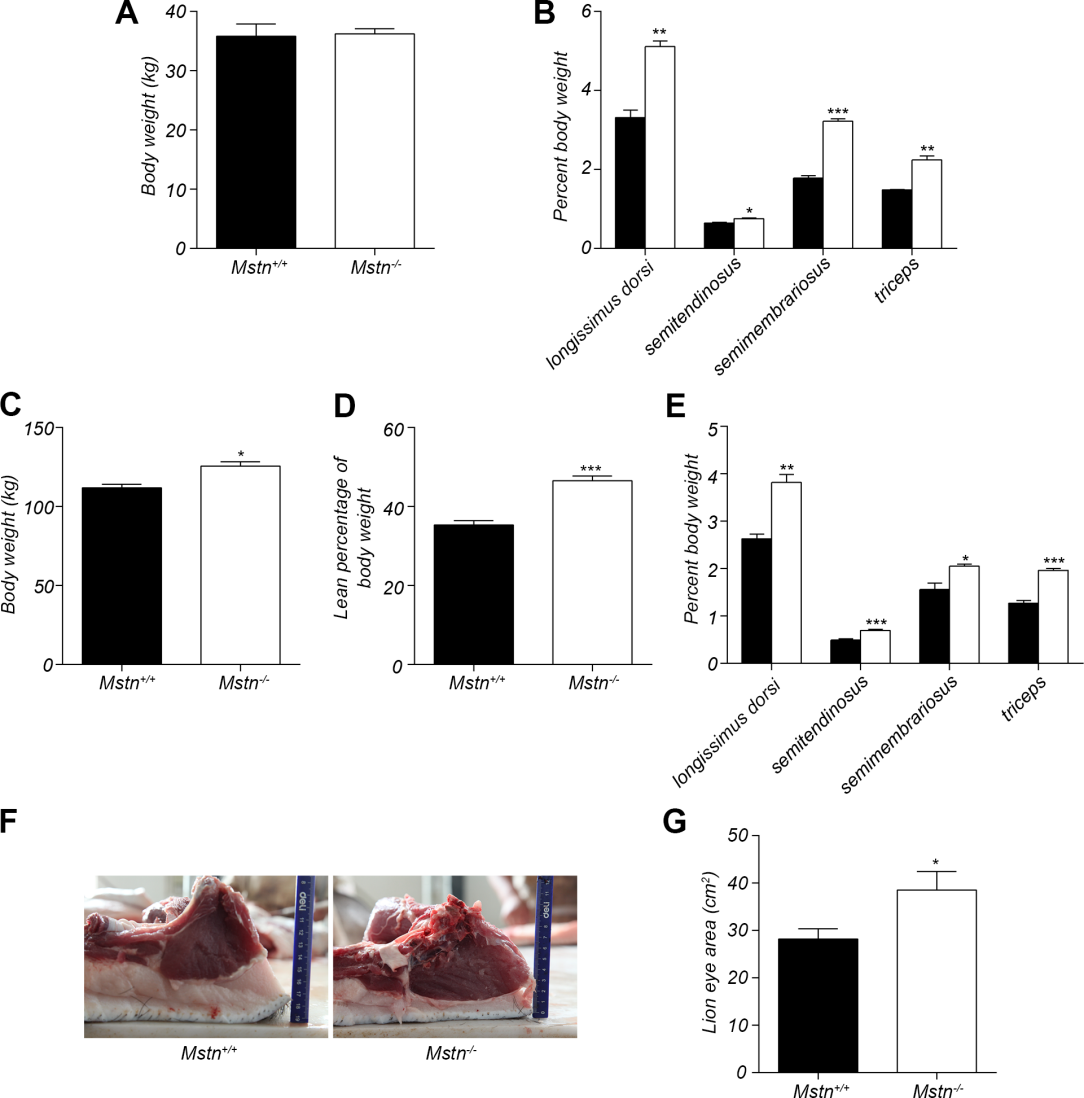
Changes in body weight and relative skeletal muscle mass (**A**) Body weight (mean ± SEM, *n* = 4 pigs) of 4-month old pigs. (**B**) Percent longissimus dorsi, semitendinosus, semimembranosus, and triceps of body weight for 4-month old pigs. Data are reported as mean ± SEM (*n* = 4). *P < 0.05, **P < 0.01, ***P < 0.001. (**C**) Body weight (mean ± SEM, *n* = 4 pigs) of 16-month old pigs. *P < 0.05, **P < 0.01, ***P < 0.001. (**D**) Percent skeletal muscle of body weight for 16-month old pigs. Data are reported as mean ± SEM (*n* = 4). *P < 0.05, **P < 0.01, ***P < 0.001. (**E**) Percent longissimus dorsi, semitendinosus, semimembranosus, and triceps of body weight of 4-month old pigs. Data are reported as mean ± SEM (*n* = 4). *P < 0.05, **P < 0.01, ***P < 0.001. (**F**) The cross section of longissimus dorsi and subcutaneous fat between 6th and 7th ribs in 16-month old pigs. (**G**) The lion eye area (mean ± SEM, *n* = 4 pigs) of longissimus dorsi between the 6th and 7th ribs. *P < 0.05, **P < 0.01, ***P < 0.001. Black bar: *Mstn*^+/+^ (wild-type) pigs; white bar: *Mstn*^−/−^ pigs.

Our lab has recently successfully generated *Mstn* ^−/−^ Meishan pigs by ZFN-editing technology [26]. In this study, the effect of myostatin deficiency on skeletal muscle and fat was further investigated at ages of 4 months and 16 months, respectively. *Mstn* ^−/−^ and wild-type Meishan pigs, fed with standard diet, were slaughtered at 4 months and 16 months, respectively. There was no significant difference in body weight between *Mstn* ^−/−^ and wild-type Meishan pigs at 4 months (Figure 1A). However, the relative mass (expressed as % of body weight) of longissimus dorsi, semitendinosus, semimembranosus and triceps all increased significantly in *Mstn* ^−/−^ Meishan pigs compared to wild-type pigs (Figure 1B). At 16 months of age, when pigs’ growth matured, the body weight of *Mstn* ^−/−^ Meishan pigs was significantly heavier than that of the wild-type pigs (Figure 1C), and the percentage of skeletal muscle mass from *Mstn* ^−/−^ Meishan pigs increased significantly (46.51% for *Mstn* ^−/−^ pigs vs 35.34% for wild-type pigs, see Figure 1D), an increase of 11.17%. Similarly, the percentages of longissimus dorsi, semitendinosus, semimembranosus and triceps of *Mstn* ^−/−^ Meishan pigs were also significantly higher than in the wild-type pigs (Figure 1E). We also isolated the longissimus dorsi muscle from 16-month-old Meishan pigs and measured their loin eye areas of carcass, and found that *Mstn* ^−/−^ Meishan pigs had much higher loin eye areas than wild-type pigs (Figure 1F and 1G). Our results show that skeletal muscle mass in *Mstn* ^−/−^ Meishan pigs increased significantly at 4 months of age and was maintained the higher level up to the adulthood age (16 months).

### A decrease in fat mass in *Mstn* ^−/−^ Meishan pigs

**Figure 2:**
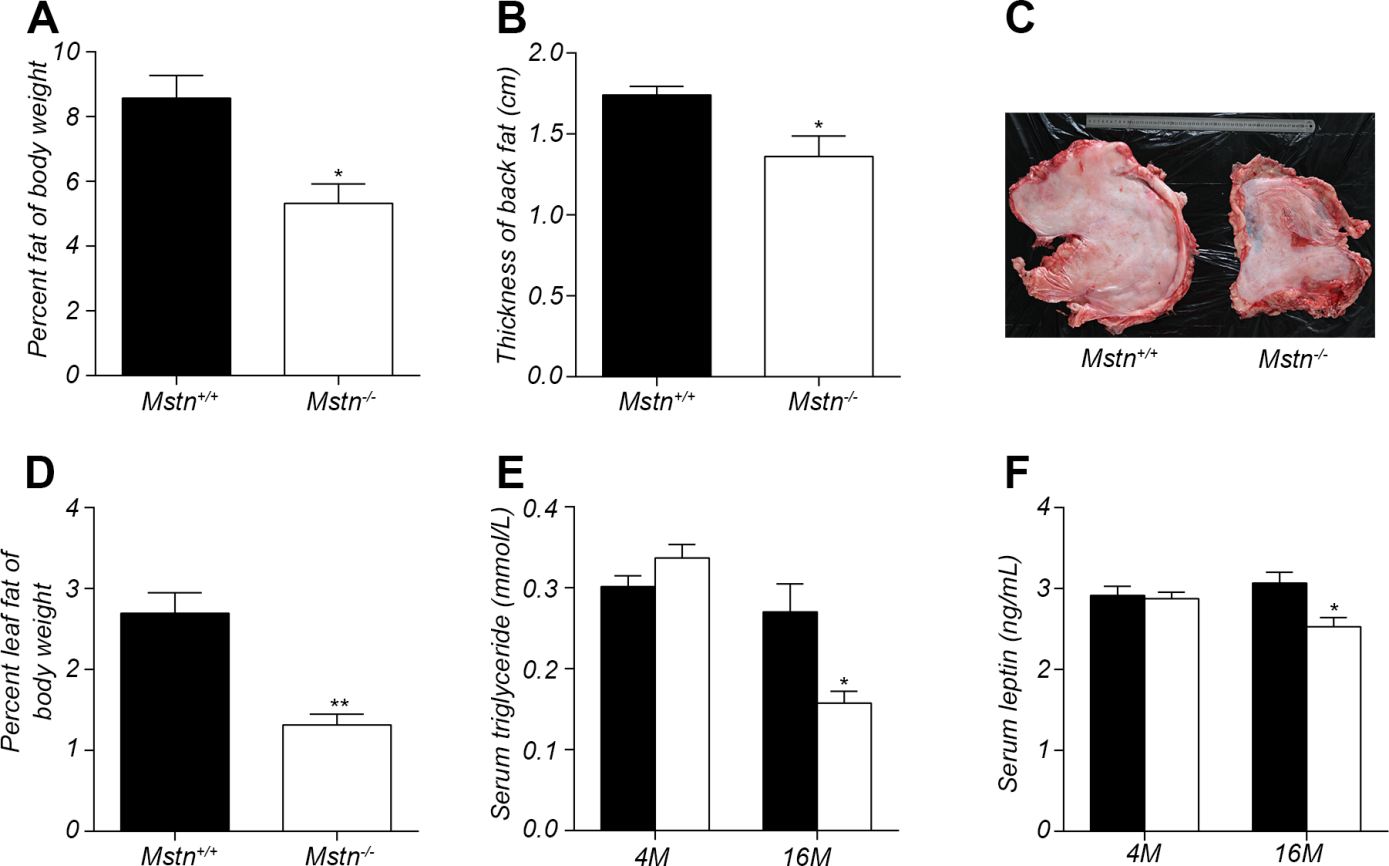
Changes in fat mass and serum levels of triglyceride and leptin (**A**) Percent subcutaneous fat of body weight for 16-month old pigs. Data are reported as mean ± SEM (*n* = 4). *P < 0.05, **P < 0.01, ***P < 0.001. (**B**) thickness of backfat between 6th and 7th ribs from 16-month old pigs. Data are reported as mean ± SEM (*n* = 4). *P < 0.05, **P < 0.01, ***P < 0.001. (**C**) Photos showing leaf fat from 16-month-old wild-type and *Mstn* ^−/−^ Meishan pigs. (**D**) Percent leaf fat of body weight for 16-month old pigs. Data are reported as mean ± SEM (*n* = 4). *P < 0.05, **P < 0.01, ***P < 0.001. (**E**) Serum level of triglyceride in 4-month old and 16-month old pigs. Data are reported as mean ± SEM (*n* = 4). *P < 0.05, **P < 0.01, ***P < 0.001. (**F**) Serum level of leptin in 4-month old and 16-month old pigs. Data are reported as mean ± SEM (*n* = 4). *P < 0.05, **P < 0.01, **P < 0.001. Black bar: *Mstn*^+/+^ (wild-type); pigs white bar: *Mstn* ^−/−^ pigs.

The excessive accumulation of white fat is the main cause of obesity, which is a serious threat to human health. The effect of myostatin deletion on fat deposition was examined in *Mstn* ^−/−^ Meishan pigs. Result in Figure 2A showed that the relative percentage of subcutaneous fat mass to body weight in *Mstn* ^−/−^ Meishan pigs is 5.324%, significantly lower than in wild-type pigs, whose value is 8.572%, a 3.248% decrease at the age of 16 months. Backfat thickness is an important index to measure subcutaneous fat deposition. Compared to wild-type pigs, *Mstn* ^−/−^ Meishan pigs have a significant reduction in backfat thickness (Figure 1F and Figure 2B). Additionally, the mass of leaf fat also decreased significantly in *Mstn* ^−/−^ Meishan pigs (1.152% in *Mstn* ^−/−^ pigs vs 2.697% in wild-type pigs), a 1.382% drop (Figure 2C–2D). Serum leptin and triglyceride levels are known to be associated with body fat deposition [27, 28]. We measured leptin and triglyceride levels in serum from 4M and 16M Meishan pigs (Figure 2E–2F), and observed no significant changes in serum leptin and triglyceride levels at 4 months between wild-type and *Mstn* ^−/−^ Meishan pigs. However, serum leptin and triglyceride levels in the 16-month-old *Mstn* ^−/−^ Meishan pigs decreased significantly (Figure 2E–2F). Further, our data show that the relative percentage of white fat and leaf fat, along with serum leptin and triglyceride levels, are all significantly reduced in adult *Mstn* ^−/−^ pigs compared to wild-type pigs.

### Increased browning of subcutaneous fat in *Mstn* ^−/−^ Meishan pigs

**Figure 3:**
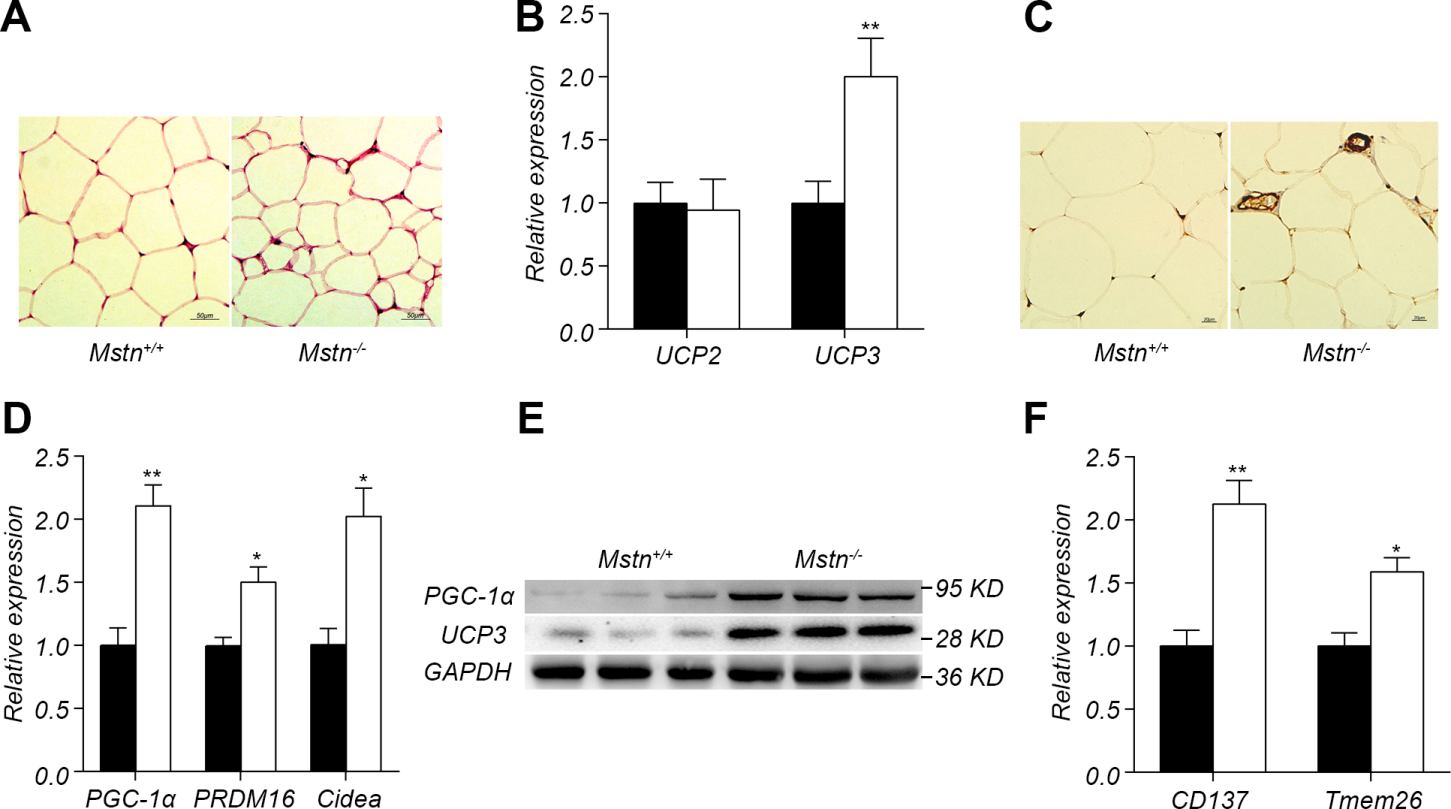
Browning of adipose fat tissue in *Mstn* ^−/−^ pigs (**A**) HE staining of dorsal subcutaneous adipose tissue between 6th and 7th ribs from 16-month old pigs. (**B**) RT-PCR analysis of UCP2 and UCP3 from dorsal subcutaneous fat from 16-month old pigs. Data are reported as mean ± SEM (*n* = 4). *P < 0.05, **P < 0.01, ***P < 0.001. (**C**) UCP3 immunohistochemical staining of dorsal subcutaneous adipose tissue between the 6th and 7th ribs from 16-month old pigs. (**D**) RT-PCR analysis of PGC1-α, PRDM16, Cidea in dorsal subcutaneous fat from 16-month old pigs. Data are reported as mean ± SEM (*n* = 4). *P < 0.05, **P < 0.01, ***P < 0.001. (**E**) Western blot of PGC1-α, UCP3 in dorsal subcutaneous fat. (**F**) RT-PCR analysis of CD137 and Tmem26 in dorsal subcutaneous fat. Data are reported as mean ± SEM (*n* = 4). *P < 0.05, **P < 0.01, ***P < 0.001. Black bar: *Mstn*^+/+^ (wild-type) pigs; white bar: *Mstn* ^−/−^ pigs.

The molecular mechanism by which fat in *Mstn* ^−/−^ mice is reduced is mainly due to the browning of white fat, which leads to an increase in fat consumption [14]. We explored the molecular mechanism of fat decrease in *Mstn* ^−/−^ Meishan pigs. HE staining results indicated that the lipid droplets in the subcutaneous adipose tissue of *Mstn* ^−/−^ Meishan pigs are smaller than in wild-type pigs (Figure 3A). The family of uncoupling protein has an important role in fat consumption. There is no significant change in the expression of UCP2 but a significant increase in the expression of UCP3 in *Mstn* ^−/−^ Meishan pigs (Figure 3B). Western blot analysis showed that UCP3 protein level increased significantly in backfat of *Mstn* ^−/−^ Meishan pigs compared with the wild-type pigs (Figure 3E). Further immunohistochemistry indicated that the UCP3 level in the subcutaneous adipocyte interstitial space of *Mstn* ^−/−^ Meishan pigs increased significantly (Figure 3C). Expression results of browning marker genes in subcutaneous adipose tissue showed that the expression of PGC-1α, PRDM16 and Cidea in *Mstn* ^−/−^ Meishan pig was significantly higher (Figure 3D). The protein level of PGC-1α in the subcutaneous fat of *Mstn* ^−/−^ Meishan pigs also increased significantly as seen in WB (Figure 3E). The expression of CD137 and Tmem26, two marker genes of beige fat, in the subcutaneous fat of *Mstn* ^−/−^ Meishan pigs was also elevated (Figure 3F). Our results suggested an increased browning of white fat in *Mstn* ^−/−^ Meishan pigs.

### Increase in insulin sensitivity induced by myostatin deletion

**Figure 4:**
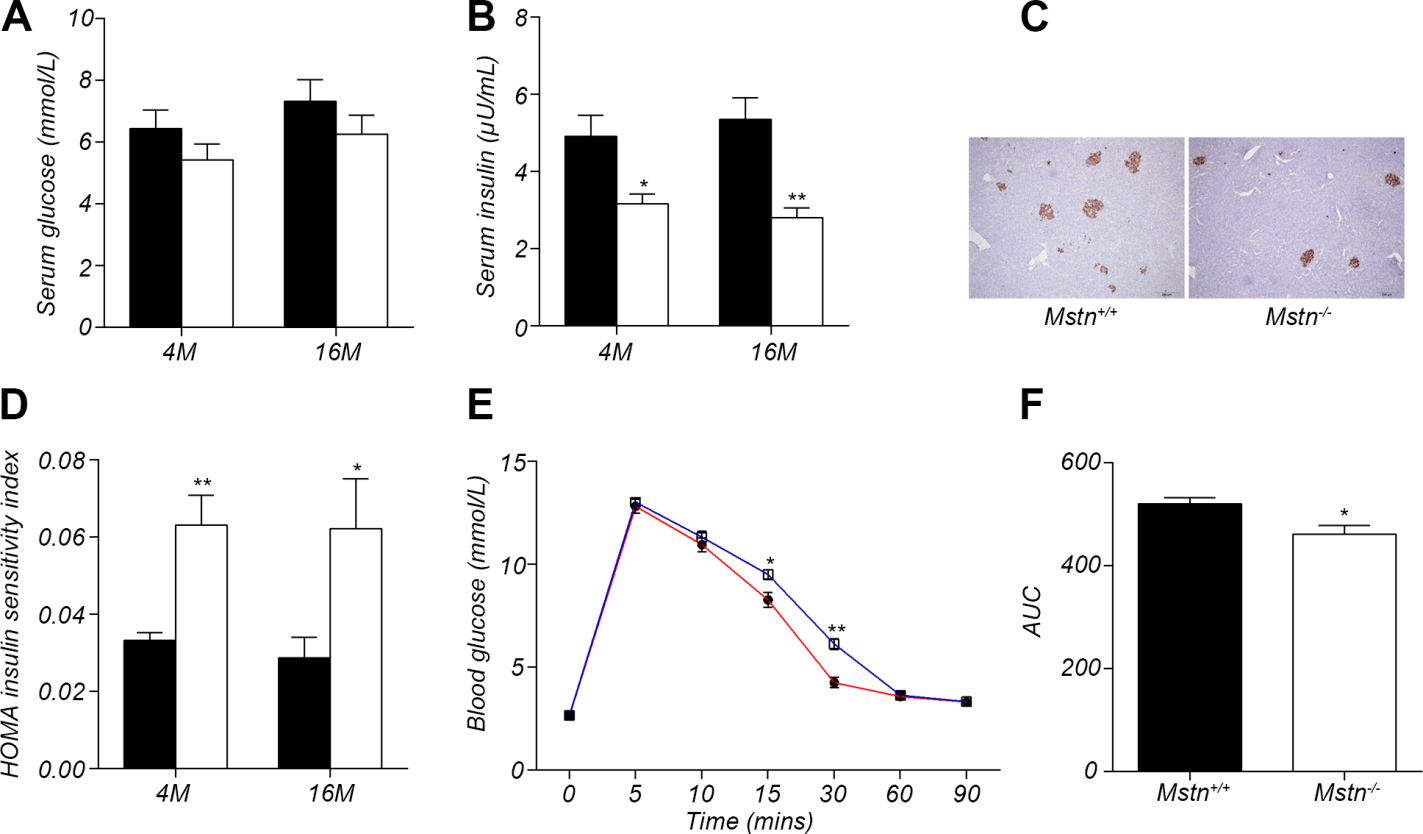
Myostatin deficiency induced increase in insulin sensitivity (**A**) Serum glucose levels in 4-month old and 16-month old *Mstn*^+/+^ (wild-type) and *Mstn* ^−/−^ pigs. Data are reported as mean ± SEM (*n* = 4). (**B**) Serum insulin levels in 4-month old and 16-month old *Mstn*^+/+^ (wild-type) and *Mstn* ^−/−^ pigs. Data are reported as mean ± SEM (*n* = 4). *P < 0.05, **P < 0.01, ***P < 0.001. (**C**) Insulin immunohistochemical staining of porcine pancreas from 4-month old *Mstn*^+/+^ (wild-type) and *Mstn* ^−/−^ pigs. (**D**) Insulin sensitivity index in 4-month old and 16-month old *Mstn*^+/+^ (wild-type) and *Mstn* ^−/−^ pigs. Data are reported as mean ± SEM (*n* = 4). *P < 0.05, **P < 0.01, ***P < 0.001. (**E**) Changes of blood glucose concentration at different time points during the glucose tolerance test. Data are reported as mean ± SEM (*n* = 4). *P < 0.05, **P < 0.01, ***P < 0.001. The blue line is for wild-type pigs, and the red line is for *Mstn* ^−/−^ pigs, (**F**) Calculated area under curve (AUC) for the glucose tolerance test. Data are reported as mean ± SEM (*n* = 4). *P < 0.05, **P < 0.01, ***P < 0.001. For A, B, D and F: Black bar: *Mstn*^+/+^ (wild-type) pigs; white bar: *Mstn* ^−/−^ pigs.

It has been reported that insulin sensitivity increased significantly in *Mstn* ^−/−^ mice compared to wild-type mice [13, 19]. However, no study has been conducted on the relationship between myostatin and insulin sensitivity in large mammals. In this study, we investigated the changes in insulin sensitivity in *Mstn* ^−/−^ Meishan pigs compared to wild-type pigs. At 4 months of age, although there is no significant difference in serum glucose level between *Mstn* ^−/−^ Meishan pigs and wild-type pigs (Figure 4A), serum insulin level was significantly lower in *Mstn* ^−/−^ Meishan pigs than in wild-type pigs (Figure 4B). The insulin sensitivity index is much greater in *Mstn* ^−/−^ Meishan pigs than in wild-type pigs (Figure 4D). We also performed insulin immunohistochemistry in pancreatic tissue and found that insulin secretion was reduced in *Mstn* ^−/−^ Meishan pigs compared to wild-type pigs (Figure 4C), which is consistent with the reduced level of serum insulin in *Mstn* ^−/−^ Meishan pigs. Similarly, at 16 months of age, serum insulin level was also significantly decreased in *Mstn* ^−/−^ Meishan pigs (Figure 4B), and the insulin sensitivity increased significantly (Figure 4D). We then performed a glucose tolerance test in 4 month-old Meishan pigs (Figure 4E). The result showed that *Mstn* ^−/−^ Meishan pigs had better glucose tolerance compared to the wild-type pigs. The values of area under curve (AUC) (Figure 4F) were significantly lower in *Mstn* ^−/−^ Meishan pigs compared to the wild-type pigs. All of these data indicate that the insulin sensitivity increased significantly in *Mstn* ^−/−^ Meishan pigs.

### Activation of insulin signaling pathway in skeletal muscle of *Mstn* ^−/−^ Meishan pigs

**Figure 5:**
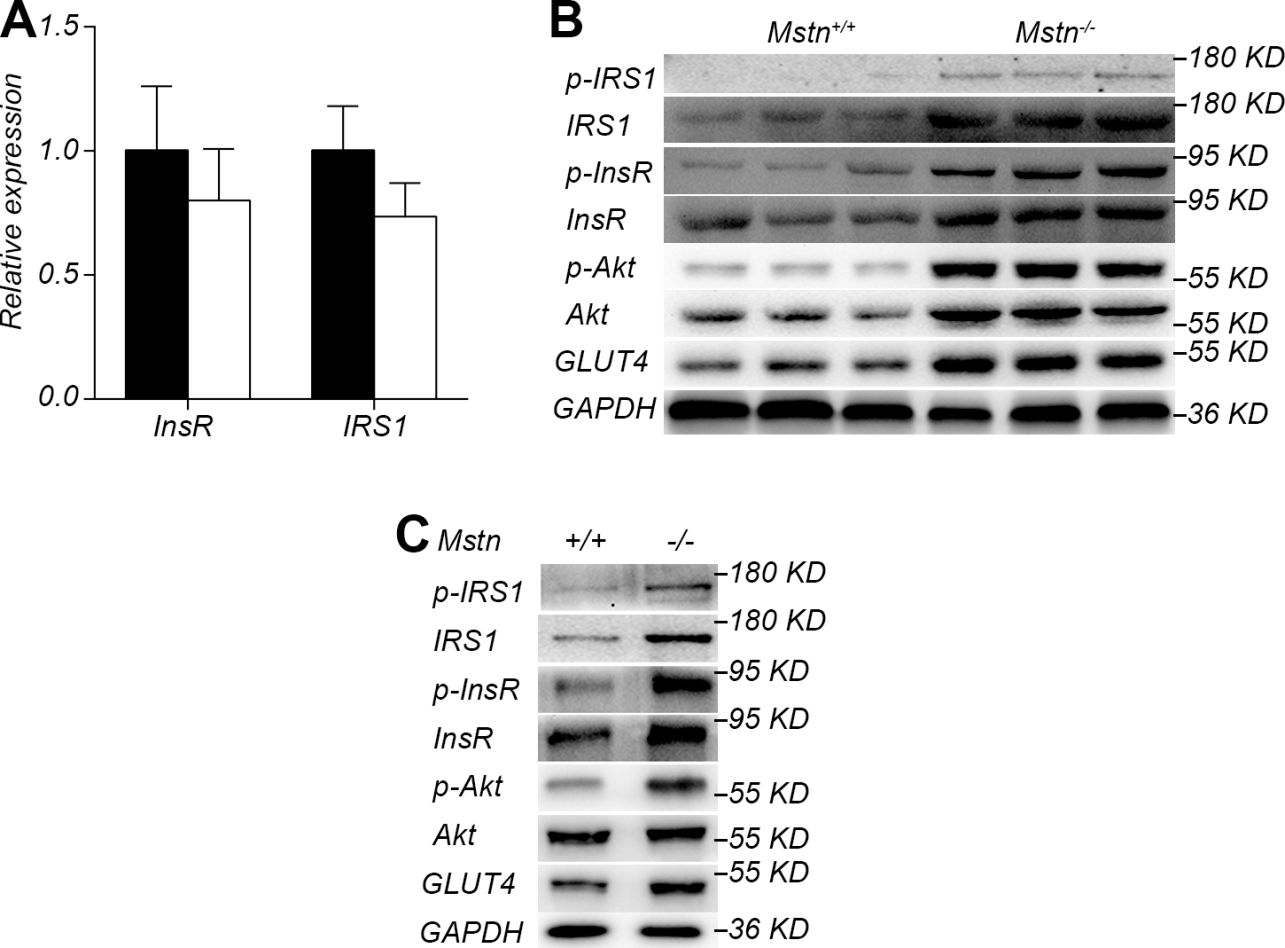
Activation of insulin signaling pathway by myostatin deficiency in skeletal muscle (**A**) RT-PCR analysis of InsR and IRS1 in longissimus dorsi from 4-month old pigs. Data are reported as mean ± SEM (*n* = 4). Black bar: *Mstn*^+/+^ (wild-type) pigs; white bar: *Mstn* ^−/−^ pigs. (**B**) Western blot of insulin signaling pathway proteins IRS1, p-IRS1, InsR, p-InsR, Akt, p-Akt, and GLUT4 in longissimus dorsi from 4-month old pigs. (**C**) Western blot of insulin signaling pathway proteins IRS1, p-IRS1, InsR, p-InsR, Akt, p-Akt, and GLUT4 of myoblasts after stimulation by insulin.

Since skeletal muscle is the main site of glucose metabolism, we investigated the changes of insulin signal pathway in longissimus dorsi. The expression of InsR and IRS1, two key proteins in the insulin signaling pathway, had no significant difference in longissimus dorsi muscle between *Mstn* ^−/−^ Meishan pigs and the wild-type pigs (Figure 5A). However, the protein levels of InsR and IRS1 in *Mstn* ^−/−^ Meishan longissimus dorsi muscle were significantly greater than that in the wild-type pigs (Figure 5B). Additionally, the phosphorylated InsR (p-InsR) and IRS1 (p-IRS1) were also significantly increased in *Mstn* ^−/−^ pigs (Figure 5B). The downstream proteins of the insulin signaling pathway, Akt, p-Akt and GLUT4, in the longissimus dorsi of *Mstn* ^−/−^ Meishan pig also increased significantly compared with the wild-type pigs. These data show that the insulin signaling pathway is activated in *Mstn* ^−/−^ Meishan pigs' skeletal muscle. Furthermore we isolated the primary myoblasts from wild-type and *Mstn* ^−/−^ Meishan pigs. We first examined the InsR expression in wild-type primary myoblasts stimulated with different concentrations of insulin. Results showed that InsR expression was at the highest level at an insulin concentration of 0.5μg/mL (Supplementary Figure 1). Thus, we did the following insulin stimulation experiments at an insulin concentration of 0.5μg/mL. Results showed the levels of InsR and IRS1 proteins, p-InsR and p-IRS1 proteins, along with the downstream proteins of the insulin signaling pathway such as p-Akt and GLUT4, all increased significantly in *Mstn* ^−/−^ Meishan porcine myoblasts (Figure 5C). These *in vitro* results, combined with the *in vivo* data described above, suggest that the inhibition of myostatin activity could increase the insulin sensitivity in skeletal muscle of Meishan pigs by activating insulin signaling pathway.

### Irisin-mediated regulation is not the only pathway for the activation of insulin signal in *Mstn* ^−/−^ skeletal muscle

**Figure 6:**
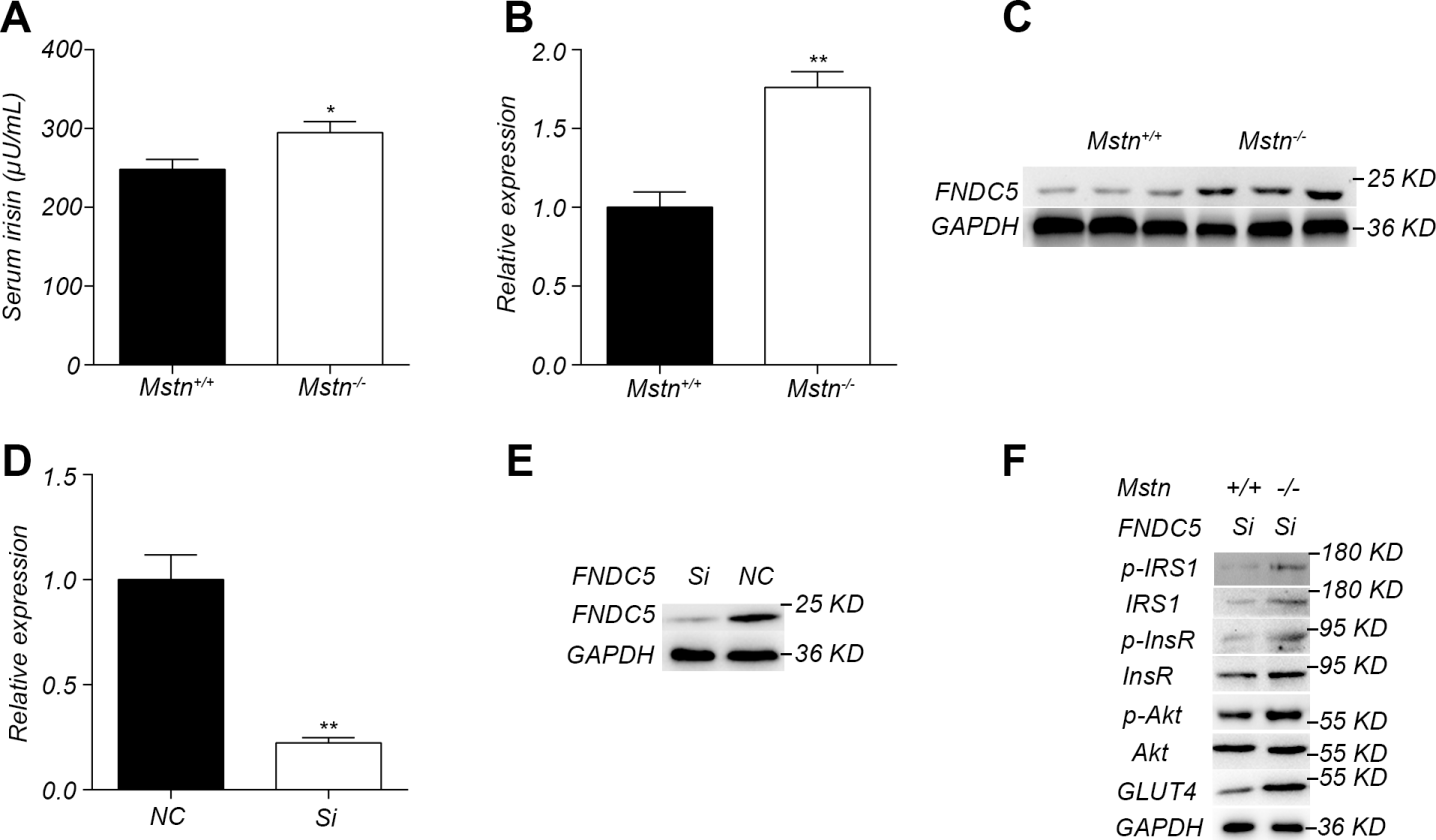
Irisin is not required for activation of insulin signaling pathway by myostatin deficiency (**A**) Serum irisin levels in 4-month old *Mstn*^+/+^ (wild-type) and *Mstn* ^−/−^ pigs. Data are reported as mean ± SEM (*n* = 4). *P < 0.05, **P < 0.01, ***P < 0.001. (**B**) RT-PCR analysis of FNDC5 in longissimus muscle from 4-month old *Mstn* ^−/−^ pigs. Data are reported as mean ± SEM (*n* = 4). *P < 0.05, **P < 0.01, ***P < 0.001. (**C**) Western blot results of FNDC5 in longissimus dorsi from 4-month old *Mstn*^+/+^ (wild-type) and *Mstn* ^−/−^ pigs. (**D**) RT-PCR analysis of FNDC5 in WT porcine myoblasts transfected with FNDC5 RNAi lentiviral vector (Si) and the control vector (NC). *P < 0.05, **P < 0.01, ***P < 0.001. (**E**) Western blot analysis of FNDC5 in WT porcine myoblasts transfected with RNAi lentiviral vector (Si) and the control vector (NC). (**F**) Western blot analysis of InsR, p-InsR, IRS1, p-IRS1, Akt, p-Akt, GLUT4, in wild type porcine and *Mstn* ^−/−^ myoblasts transfected with RNAi lentiviral vector (Si). For A, B and D: Black bar: *Mstn*^+/+^ (wild-type) pigs; white bar: *Mstn* ^−/−^ pigs.

Irisin is a new muscle factor that plays an important role in the regulation of insulin sensitivity. There had been reports that irisin level was significantly increased in *Mstn* ^−/−^ mice skeletal muscle [14, 24]. We further investigated the relationship between irisin and myostatin in the activation of insulin signaling pathway in skeletal muscle. Serum irisin level was significantly higher in the 4-month-old *Mstn* ^−/−^ Meishan pigs compared to the wild-type pigs (Figure 6A). Similarly, the expression and protein level of FNDC5, a precursor protein of irisin, also increased significantly in *Mstn* ^−/−^ skeletal muscle (Figure 6B, 6C). Therefore, we speculate that knockout of *Mstn* leads to an increase of irisin synthesis in skeletal muscle and thus results in the activation of insulin signaling pathway. We then investigated if there exists an alternative pathway in which irisin is not required for myostatin regulating insulin sensitivity in skeletal muscle. So FNDC5 interfering RNAs were first transfected in primary porcine myoblasts by a lentiviral vector. The lentiviral transfection efficiency was confirmed high (Supplementary Figure 2). As expected, FNDC5 expression at both transcriptional and translational levels was significantly reduced in FNDC5 SiRNA transfected myoblasts (Figure 6D, 6E). However, insulin stimulation experiments showed that the activation of insulin signal pathway in primary *Mstn* ^−/−^ myoblasts was not abolished compared to the wild-type myoblasts (Figure 6F). These data indicate that the inhibition of irisin expression cannot abolish the activation of insulin signaling pathway by myostatin deletion in skeletal muscle, implying that irisin-mediated regulation is not the only pathway for the activation of insulin signal in *Mstn* ^−/−^ skeletal muscle.

## DISCUSSION

Animal models play an important role in human disease studies, and thus the selection of animals in this field is critical. Mouse is one of the most widely used animals due to its high reproduction rate, short life cycle, low cost, and easy experimental manipulation. However, mouse has its own disadvantages, which include huge differences in genome sequences and physiological structures between mouse and human. Thus, mouse is not an ideal animal to study human diseases. Recently, porcine genome sequences have been completed and published, and it was noted there is a very high similarity to human genome [25]. Additionally, pig has very similar physiological structure to human and high reproduction rate. These characteristics make pig an ideal model animal to study human diseases. Most importantly, pig has advantages in the research area of obesity and type 2 diabetes [29–31]. Like human, pigs eat all kinds of food including vegetables and meat. The sizes of fat cells as well as the fat distribution are very similar between pig and human. Both pig and human contain subcutaneous fat and visceral fat. The size, shape, and location of pancreas are also similar between pig and human. The size of the islets as well as the proportion and distribution of the different endocrine cell types is also very similar in pig and human. Porcine insulin differs from human insulin in only one amino acid at position 30 of the B-chain and has been used to treat diabetic patients. Pig and human also have similar metabolism system and cardiovascular system. Therefore, use of pig as an animal model is very important in studying human obesity and type 2 diabetes.

Myostatin is a muscular factor that negatively regulates the growth and development of skeletal muscle. In large animals such as bovines [5, 6], sheep [32], dogs [33], and humans [34], it has been found that natural mutations of myostatin result in skeletal muscle hypertrophy. Our results in this study demonstrated that the skeletal muscle mass of *Mstn* ^−/−^ Meishan pig was significantly higher than that of wild-type pigs at ages of 4 months and 16 months.

Adipose tissue is divided into white fat, brown fat, and beige fat [35, 36]. The main function of white adipose tissue is to store energy, while the brown fat is to burn stored energy. Beige fat is a form of browning of white fat and has the function of brown fat. In mice, UCP1 is a brown fat marker gene, it can consume the stored energy in the form of heat. When myostatin activity is inhibited in mice, the expression of UCP1 in subcutaneous adipose tissue increased significantly, and the expression of PGC-1α, PRDM16, and Cidea in white fat also increased significantly, which in turn induced more consumption of white fat [14]. It is known that porcine *UCP1* gene is disrupted and cannot form active UCP1 protein [37]. UCP2 and UCP3 are also members of the uncoupling protein family, and they can also stimulate fat consumption [38, 39]. J Jia at el [40] reported that feeding pigs with a low protein diet leads to an increase in thermogenesis and a significant increase in expression of UCP2 and UCP3 in skeletal muscle and fat tissue. *In vivo*, ATP synthesis increased significantly in primary skeletal muscle cells isolated from UCP3 knockout mice without any increase in TCA cycle flux rate, implying an increased degree of mitochondrial energy coupling [41]. All these observations indicate that UCP3 can generate heat by enhancing energy consumption. On the other hand, it was reported that no changes in UCP3 expression levels were observed when pigs were raised under extremely cold environment [42]. The energy expenditure of whole body in UCP3 knockout mice did not change [41]. In this study, the expression of UCP3 in the subcutaneous fat was significantly higher in *Mstn* ^−/−^ Meishan pigs compared to wild-type pigs. There are still disputes on the roles of porcine UCP3 in heat production and energy consumption, and therefore more research is required in this area.

Type 2 diabetes is a worldwide disease that posts a serious threat to human health. Insulin resistance is the leading cause of type 2 diabetes. So far, there was no study to report the relationship between myostatin and insulin sensitivity in large mammals. In this study, we observed that *Mstn* ^−/−^ Meishan pigs have significantly higher insulin sensitivity than wild-type pigs, which is consistent with results reported in *Mstn* ^−/−^ mice [13, 23]. There are a few reports on the molecular mechanism by which myostatin regulates insulin sensitivity. InsR and IRS1 are the key proteins of the insulin signal pathway, and inhibition of mouse InsR and IRS1 activity can lead to insulin resistance in mice [43–46]. Bonala et al. [8] have reported that myostatin can promote IRS1 ubiquitination and its degradation in mouse skeletal muscle, which in turn inhibit the activation of insulin signaling pathway, leading to insulin resistance. Our results showed that levels of InsR and IRS1 proteins in skeletal muscle from *Mstn* ^−/−^ Meishan pigs were significantly increased, which is the cause of activated insulin signaling. Our study is the first report that myostatin deficiency can activate the insulin signal and increase insulin sensitivity in large animals.

Irisin is an active secretory protein that is produced by the cleavage of its precursor protein FNDC5 [47]. Irisin protein is mainly synthesized in the skeletal muscle, and like myostatin, it is highly conserved among a variety of species. Irisin can significantly improve insulin sensitivity in mice [31, 48]. Previous studies show that the increases in insulin sensitivity and expression of FNDC5 in skeletal muscle were observed in *Mstn* ^−/−^ mice [14, 24]. In our current study, we demonstrated that the activation of insulin signal pathway in primary *Mstn* ^−/−^ myoblasts was not completely abolished by FNDC5 SiRNA, implying that irisin-mediated regulation is not the only pathway for the activation of insulin signal in *Mstn* ^−/−^ skeletal muscle. More studies are needed to further explore the molecular mechanism by which the insulin sensitivity is regulated by myostatin.

In summary, our results demonstrate that the increased browning process and fat consumption are the key reasons for a decreased fat deposition in *Mstn* ^−/−^ Meishan pigs. The myostatin deficiency also resulted in a significant increase in insulin sensitivity and the activation of insulin signaling pathway in large animals. It is expected that inhibition of myostatin function can be an effective alternative approach in enhancing insulin sensitivity and thus for potential prevention and treatment of type 2 diabetes.

## MATERIALS AND METHODS

### Animals

*Mstn* ^−/−^ pigs were generated as previously described [26]. All experimental protocols related to animal work were approved by the Institute of Animal Sciences, Chinese Academy of Agricultural Sciences, Beijing, China. All experimental pigs, both wild-type (*Mstn*^+/+^) and *Mstn* ^−/−^, received adequate housing, feed (standard diet), access to water and bedding.

### Serum measurements

The levels of serum glucose and triglyceride (TG) were determined with Biochemical analyzer (TBA-120FR). Serum leptin was measured using the Porcine Leptin ELISA Kit (Elabscience, E-EL-P0063), and serum insulin concentration was measured with Porcine Insulin ELISA Kit (Lanpaibio, hj-C14701). Serum irisin level was determined by Irisin Competitive ELISA Kit (Biovision, K4761-100).

### Haematoxylin and eosin staining and immunohistochemistry

Subcutaneous adipose tissue and pancreas were fixed in 4% paraformaldehyde at room temperature for 48h, followed by embedding in paraffin. Each fixed tissue was then sliced to 5μm in thickness to make slides, followed by haematoxylin and eosin (HE) staining and immunohistochemical staining. Immunohistochemistry was performed according to standard procedures to stain uncoupling protein 3 (UCP3) and insulin using anti-UCP3 antibody ((AB10985, Abcam) and anti-insulin antibody (AB195956, Abcam).

### RNA extraction, cDNA synthesis and real time PCR (RT-PCR)

RNA extraction, cDNA synthesis and real time PCR were performed as previously described [26]. RT-PCR primers were shown in Supplementary Table 1. Porcine TATA-binding protein 1 (*TBP1*) gene was used as an internal control [49] to normalize the RT-PCR efficiency and to quantify the expression of the genes in wild-type and *Mstn* ^−/−^ pigs.

### Protein extraction and Western blot

Total protein from the muscle and adipose tissue was extracted with T-PER Tissue Protein Extraction Reagent (Thermo, 78510), and total cellular protein was extracted with M-PER Mammalian Protein Extraction Reagent (Thermo, 78501). Protease inhibitor cocktail tablets (Roche, 04693159001) and phosphatase inhibitor cocktail tablets (Roche, 04906837001) were added to T-PER Tissue Protein Extraction Reagent and M-PER Mammalian Protein Extraction Reagent prior to use. Total protein concentration was determined using a Micro BCA™ Protein Assay Kit (Thermo, 23235). Each protein sample was loaded in equal amount and then separated by 10% SDS PAGE. Following transfer of protein from gel to NC membrane (Merck Millipore, HATF00010) and blocked with 5% BSA (Amresco, 9048-46-8) for 2h, Western blot was performed using standard method for the following proteins with corresponding detection antibodies (in brackets): IRS1 [Cell Signal Technology (CST), 2825], phosphorylated IRS1 (p-IRS1) (CST, 2831), insulin receptor (InsR) (CST, 3025), p-InsR (CST, 3023), serine/threonine kinase 1 (Akt) (CST, 9272), p-Akt (CST, 9271), facilitated glucose transporter 4 (GLUT4) (CST, 2213), PGC-1α (Santa, sc-13067), UCP3 (Abcam, ab10985), fibronectin type III domain containing 5 (FNDC5) (Abcam, ab174833), anti-mouse secondary antibody (CST, 7076), anti-rabbit secondary antibody (CST, 7074). Glyceraldehyde-3-phosphate dehydrogenase (GAPDH) (CST, 2118) was used as an internal reference in Western blot. SuperSignal West Pico chemiluminescent substrate (Thermo, 34080) was used to develop color band.

### Glucose tolerance test

50% glucose (1.2 ml/kg of body weight) was injected into the ear vein to each of the 4-month-old Meishan pigs. Level of blood glucose was measured at different time points after injection using a blood glucose meter (SANNUO).

### Isolation of primary skeletal myoblasts

Newly born piglets were euthanized and soaked in 75% alcohol for 5min. The longissimus dorsi muscle was separated and cut into pieces, followed by adding 0.1% collagenase type I (GIBCO, 17100017) and incubation at 37°C for 2 hours. Then 0.25% trypsin was added and incubated for 5 min to release cells, followed by adding 20% FBS (GIBCO, 10099-141), DMEM/F12 (Hyclone, SH30023.01B), and 1% penicillin-streptomycin to stop the digestion process. Cells were then filtered (70 μm). The filtrate was centrifuged to remove supernatant, and the cell pellet was then resuspended in culture medium containing 20% FBS, DMEM/F12, 1% penicillin-streptomycin. Cells were then transferred into 60mm dish, incubated at 37°C and 5% CO_2_ for 2 hours, the supernatant containing myoblasts was then transferred into 35mm dish, incubated at 37°C and 5% CO_2_, and changed culture medium once every 2 days.

### Insulin stimulation

Insulin stimulation experiments were performed as described by Bonala *et al*. (8) using the primary myoblasts at a cell density of 10000 cells/cm^2^ in DMEM/F12 medium containing 20% FBS and 1% penicillin-streptomycin.

### Lentiviral-mediated transduction

The FNDC5 interfering RNA sequence (GCGATGCACAACTTTGCAAGT) was designed by GenePharma. Vector construction and lentiviral packaging were also performed by GenePharma. The primary myoblasts were inoculated in T25 flask. When cell confluency reached 50%, DMEM/F12 medium containing 20% FBS was added along with 10μL of virus stock and 5μg/mL of Polybrene. Cells were then incubated at 37°C and 5% CO_2_ for 24 hours. At 24h hours and 48 hours, fresh DMEM/F12 medium containing 20% FBS was replaced, respectively. Then DMEM/F12 medium containing 20% FBS and 5 μg/mL of Puromycin was added. Cell culture was continued with fresh medium being changed every three days until stable expression of red fluorescent protein was observed.

### Data analysis

All data were analyzed by using unpaired 2-tailed Student's *t* tests (*P <* 0.05).

## SUPPLEMENTARY MATERIALS FIGURES AND TABLES



## Abbreviations

Mstn: Myostatin
GDF8: Growth/differentiation factor 8
TGFβ: Transforming growth factor beta superfamily
WT: Wild-type
UCP: Uncoupling protein
PGC-1α: Peroxisome proliferative activated receptor, gamma, coactivator 1 alpha
PRDM16: PR domain containing 16
Cidea: Cell death-inducing DNA fragmentation factor, alpha subunit-like effector A
CD137: Tumor necrosis factor receptor superfamily member 9
Tmem26: Transmembrane protein 26
IRS1: Insulin receptor substrate 1
p-IRS1: Phosphorylated insulin receptor substrate 1
ZFN: Zinc-fingers Nuclease
AUC: Area under curve
InsR: Insulin receptor
p-InsR: Phosphorylated insulin receptor
Akt: Serine/threonine kinase
p-Akt: Phosphorylated serine/threonine kinase
GLUT4: Facilitated glucose transporter member 4
HE staining: Haematoxylin and eosin staining
RT-PCR: Real time PCR
TBP1: TATA-binding protein 1
FBS: Fetal bovine serum
DMEM/F12: Dulbecco›s Modified Eagle Media: Nutrient Mixture F-12
TCA: tricarboxylic acid
