# Supplementary material for: Loss-of-function myostatin mutation increases insulin sensitivity and browning of white fat in Meishan pigs
Source: Oncotarget. 2017 Apr 4;8(21):34911–22. doi: 10.18632/oncotarget.16822 (PMC5471021; doi:10.18632/oncotarget.16822)
Supplement: Supplementary file 1 [file oncotarget-08-34911-s001.pdf]

Loss-of-function myostatin mutation increases insulin sensitivity and browning of white fat in Meishan pigs

Supplementary Materials

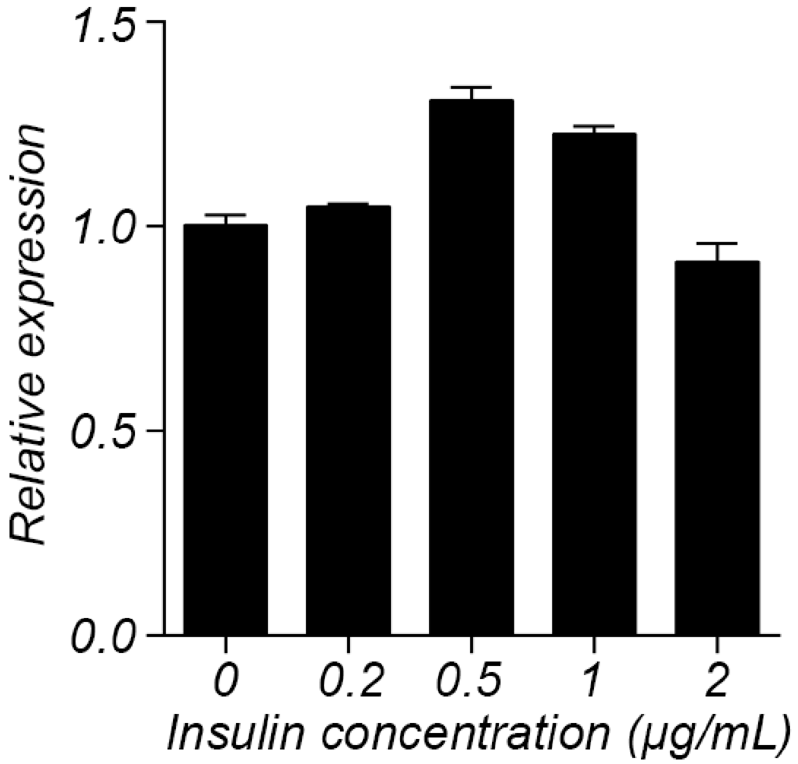

Supplementary Figure 1: RT-PCR analysis of InsR in WT porcine primary myoblasts stimulated with different insulin concentrations (μg/mL).

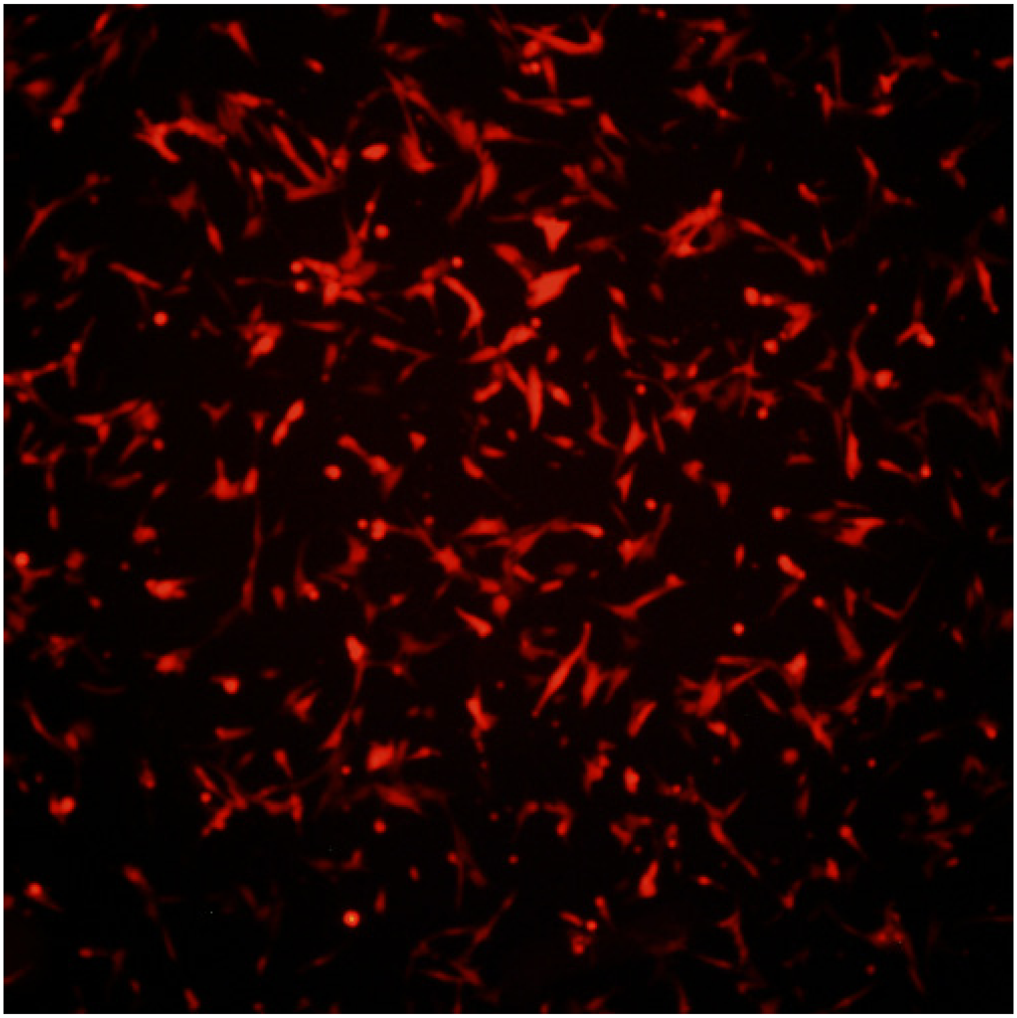

Supplementary Figure 2: Transfection efficiency of FNDC5 interfering RNA vector in porcine myoblasts. Red color indicates that myoblasts have been transfected with the vector.

Supplementary Table 1: Primers for RT-PCR analysis in meishan pigs

Genes	Forward primers	Reverse primers
UCP2	AGTGTGAGACCTGACGAAGC	CCTTTCTCCCTGGATCTGC
UCP3	CAACAGGAAGTACAGCGGGA	CACCATCTCGGCACAGTTCA
PGC-1α	CACCAGCCAACACTCAGCTA	GAGGTGCACTTGTCTCTGCT
PRDM16	TACACGTGCAGGTACTGTGG	GAGGTGTCTGTCCAGGTTGG
Cidea	GGGAGATAAGGGTCAGCGTG	AAGCAGAGATGAAGAGGAAGCA
CD137	AAACAACCGTTTCTGAAGCCAG	TCAAGAGAGTCCCAGCACCT
Tmem26	TCTACCCATTGGAGGTGGGA	GCAGGGCTCTTCCTCACATTT
InsR	CTGCGTCACTTCACTGGCTA	CCTCATCACCGTATCGTCGG
IRS1	AGAGGACCGTCAGTAGCTCA	CACGATAGAGAGCGTGCAGT
FNDC5	TGCAGGCCATCTCCATTCAG	ATATTGGCGGCAGAAGAGGG
TBP1	AACAGTTCAGTAGTTATGAGCCAGA	AGATGTTCTCAAACGCTTCG
